# SalfMix: A Novel Single Image-Based Data Augmentation Technique Using a Saliency Map

**DOI:** 10.3390/s21248444

**Published:** 2021-12-17

**Authors:** Jaehyeop Choi, Chaehyeon Lee, Donggyu Lee, Heechul Jung

**Affiliations:** Department of Artificial Intelligence, Kyungpook National University, Daegu 41566, Korea; jaebb95@knu.ac.kr (J.C.); 123456ccdd@knu.ac.kr (C.L.); dglee@knu.ac.kr (D.L.)

**Keywords:** deep learning, data augmentation, convolutional neural network (CNN), image classification

## Abstract

Modern data augmentation strategies such as Cutout, Mixup, and CutMix, have achieved good performance in image recognition tasks. Particularly, the data augmentation approaches, such as Mixup and CutMix, that mix two images to generate a mixed training image, could generalize convolutional neural networks better than single image-based data augmentation approaches such as Cutout. We focus on the fact that the mixed image can improve generalization ability, and we wondered if it would be effective to apply it to a single image. Consequently, we propose a new data augmentation method to produce a self-mixed image based on a saliency map, called SalfMix. Furthermore, we combined SalfMix with state-of-the-art two images-based approaches, such as Mixup, SaliencyMix, and CutMix, to increase the performance, called HybridMix. The proposed SalfMix achieved better accuracies than Cutout, and HybridMix achieved state-of-the-art performance on three classification datasets: CIFAR-10, CIFAR-100, and TinyImageNet-200. Furthermore, HybridMix achieved the best accuracy in object detection tasks on the VOC dataset, in terms of mean average precision.

## 1. Introduction

**Figure 1:**
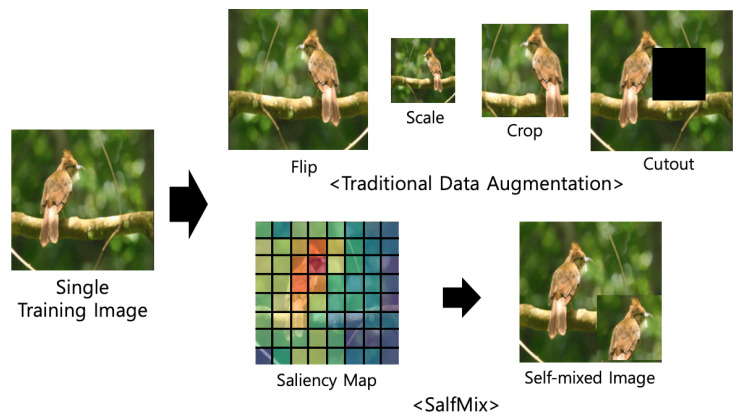
Conceptual comparison between our proposed SalfMix method and other single image-based data augmentation methods. Our proposed SalfMix transforms a single image into a self-mixed image guided by a saliency map.

Deep learning has achieved remarkable performances in various computer vision tasks such as image classification [1,2,3,4], segmentation [5,6], detection [7,8,9,10,11], and image quality assessment [12]. Generally, deep neural networks (DNNs) require large training data to achieve high performance. Data augmentation techniques can increase the limited size of training data and are important elements in the training process of DNNs to improve their generalization performances. Data augmentation techniques have been used to train AlexNet [13], and geometric data augmentation approaches have been used to reduce Top-5 error rates of ImageNet classification tasks, such as flip, rotation, crop, and translation [13,14]. In 2014, VGG neural networks were proposed, and the scale jittering data augmentation technique was introduced by [15]. The Cutout method, which is a representative data augmentation approach, performs regional dropout, where pixel values of a randomly selected region of an input image are removed [16]. Regional dropout approaches have shown better recognition rates than previous geometric transformation strategies [16,17]. These data augmentation approaches are performed on a single image, as shown in Figure 1.

In the recent data augmentation studies, two training images are selected and mixed during network training, and mixed images are used for training a convolutional neural network (CNN), such as Mixup [18] and CutMix [19]. These techniques further improve generalization performance than traditional single image-based approaches. Most recent research works such as SaliencyMix [20], PuzzleMix [21], ResizeMix [22], and SnapMix [23] focus on the mixing of two images for data augmentation. Especially, when CutMix mixes images, random patches are cut and pasted on other images; however, saliency-guided approaches have recently been proposed and achieve better performances than the original CutMix [20,21,23]. However, when using saliency-guided approaches [20,23], there are still cases where the important feature of the image is lost when the salient regions of the two images to be mixed overlap at the same location. because they do not increase important features in the image itself.

In this paper, we present two kinds of data augmentation strategies. The SalfMix uses a saliency map for self-guidance. It is important to extract important features that can predict the class of input images. The saliency map represents the importance of the image in training and can be utilized to augment the image including more important features. It produces a self-mixed image based on a single training image. The performance of the approach outperforms the Cutout method, which is one of the state-of-the-art single image-based data augmentation techniques [16] (e.g., 19.89%, SalfMix VS 21.46%, Cutout error rates in CIFAR-100 with PreActResNet-101). Additionally, we prove that the SalfMix data augmentation technique could increase the state-of-the-art performance of two images-based data augmentation approaches, such as Mixup, SaliencyMix, and CutMix, by linearly combining two approaches without any modification. We call it HybridMix. HybridMix does not lost important features even if the salient regions of the two images to be mixed overlap at the same location, because it applies SalfMix together. We summarize the key differences among state-of-the-art data augmentation techniques, including our proposed approaches, in Table 1.

The contributions of this paper can be summarized as follows:

- A new single image-based data augmentation method SalfMix uses a self-guidance method finding import region and conduct meaningful mixing in a single image.
- The proposed SalfMix can replace the least salient region with most salient region considering spatial importance from a saliency map.
- We propose HybridMix, which simply combines SalfMix and two images-based approaches, and shows the best performance among state-of-the-art data augmentation methods.

## 2. Related Work

### 2.1. Randomly Patched Data Augmentation

**Cutout and Random regional dropout** [16,17] are techniques that remove random rectangular parts of an input image and fill it with zeros or random values. Unlike the dropout [25], the approaches remove regionally continuous parts in the input layer. These techniques improve the generalization performance of a model in classification and object localization tasks.  

**Mixup** [18] is data augmentation technique to mix two random images. Mixup blends the images and their labels using linear interpolation. It improved the classification performance of a model and demonstrated its robustness to adversarial attacks through training using mixed images. However, Mixup produces an image revealing locally ambiguous and unnatural characteristics that can confuse localization. 

**CutMix** [19] was published in 2019 by supplementing Cutout and Mixup. CutMix is a data augmentation strategy that uses patches that are cut and pasted on images. Image patches are randomly selected, and image labels are mixed. Compared to Cutout, this strategy minimizes information loss by replacing the removed areas with patches from other images and provides good performance in various image recognition tasks. 

**ResizeMix** [22] was published in 2020 to preserve more substantial object information than CutMix. ResizeMix proposed a method that mixes not a cutting-based image but resized image. The resized image might have the original object’s identities, and this might be helpful for improving performance in image classification and object detection compared to CutMix. 

**Self-Augmentation** [24] firstly proposed a self-mix method that cuts a random patch from an image and pastes into the same image. The goal of the research is to improve generalization ability in a few-shot learning scenario. They did not show improvements in large-scale image recognition tasks like classification, segmentation and object detection. Although our approach looks similar tothis method, it’s not because we use a saliency map containing importance for each pixel and extend it to a form mixed with two images-based data augmentation.

### 2.2. Saliency-Guided Data Augmentation

**PuzzleMix** [21] significantly improves the performance of the existing data augmentation technique Mixup using saliency information. In other words, the novel Mixup that explicitly utilizes saliency information was proposed, and achieves better generalization and the adversarial robustness than other Mixup methods.  

**SnapMix** [23] is a semantic proportional mixing method using a class activation map [26]. In CutMix, noisy labels can occur because CutMix may select a random region that does not contain the object. SnapMix determines the label ratio based on semantic percentage maps. Therefore, the noisy labels can be prevented. The proposed SnapMix achieves the state-of-the-art performance for fine-grained recognition. 

**SaliencyMix** [20] uses the saliency detection method which is a hand-crafted saliency map designed for fast human detection in a scene [27]. SaliencyMix conducted a study on how to mix patches based on the saliency map. Finally, SaliencyMix achieves the state-of-the-art performance in various image classification tasks, such as CIFAR-10, 100, and ImageNet datasets.

## 3. Motivation and Problem Statement

We denote T as a data augmentation function. $T_{s}\left(x_{1}\right)$ and $T_{t}\left(x_{1},y_{1},x_{2},y_{2}\right)$ represent single image-based and two images-based data augmentation functions, respectively, with arbitrary training images $x_{1}$, $x_{2}$ and their labels $y_{1}$, $y_{2}$. The functions can be combined as follows:

(1) $$\tilde{x}\leftarrow T_{t}\left(T_{s}\left(x_{1}\right),y_{1},T_{s}\left(x_{2}\right),y_{2}\right).$$

$\tilde{x}$ is used for training DNNs. Based on the equation, single image-based data augmentation techniques such as Cutout can be integrated with two images-based approaches. Currently, various research studies focus on developing two images-based data augmentation techniques. However, we believe that the single image-based data augmentation technique still plays a big role to improve the generalization performance of DNNs. Our goal in this research is to develop a new single image-based data augmentation technique, and the developed algorithm may synergize with traditional two images-based data augmentation approaches (e.g., Mixup, SaliencyMix, CutMix).

## 4. Proposed Method

### 4.1. Saliency-Guided Data Augmentation

**Figure 2:**
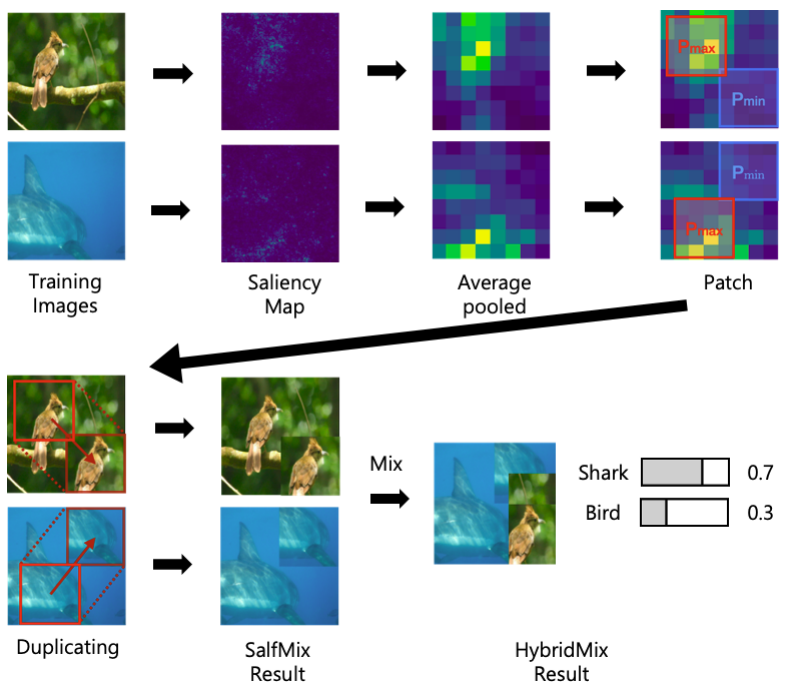
Overall process of the proposed approach. Self-mixed images are created by replacing the least salient region with the most salient region. Then, a two images-based data augmentation approach is performed to create HybridMix result.

To develop a new single image-based data augmentation technique, we adopt a saliency map-based self-guidance method. The self-guidance method guides which part of an image should be removed or duplicated. Based on the self-guidance, we generate combined training samples for every epoch, which are used as training data. Figure 2 shows the overall process of the proposed method. First, two images are randomly selected from a training dataset. Then, a saliency map for each image is computed based on the trained network. The most salient region is cropped and pasted to the least salient region in the same image. This step is repeated for the other selected image. Using the two synthesized images, two images-based data augmentation is then used.

### 4.2. Saliency Map Extraction

In this step, we would like to calculate a spatial importance score of a given image for self-guidance. The least important region will be removed and replaced using the most important region. A saliency map is one of the choices to compute the spatial importance score. We compute gradients to generate the saliency map as follows [28]:

(2) $$g=\frac{\partial L(f(x,\theta ),y)}{\partial x},$$

where the L(·) is a cross-entropy loss function, and $x\in R^{W\times H\times 3}$ and *y* denote an input image and its true label, respectively. *W* and *H* are the width and height of an input image, respectively. f:x→y denotes a CNN function and θ is set of parameters of *f*. The loss value is obtained by passing through the CNN function. Next, we proceed with the backpropagation with the obtained loss value and obtain the gradient value *g*.

Based on the $g\in R^{\times W\times H\times 3}$, we create a saliency map $M\in R^{W\times H}$ that indicates the importance of pixels in the input image. g(i,j,c) is the gradient value of the c-th channel (e.g., RGB channel), *i*-th column and *j*-th row corresponding to the input image. The (i,j)-th pixel value of the saliency map is the maximum absolute value of the corresponding pixel values on all channels:

(3) $$M_{ij}=\max\left(|g\left(i,j,0\right)|,|g\left(i,j,1\right)|,|g\left(i,j,2\right)|\right),$$

where the numbers 0,1,2 indicate the red, green and blue channels, respectively.

### 4.3. SalfMix

The next step is to perform average pooling to the saliency map. Average pooling is used to determine the importance of a region rather than a pixel. Then, *M* is transformed as illustrated in the average-pooled of Figure 2. We denote it as $\bar{M}$. In Figure 2, the second image, which name is the saliency map, means *M* and the third image, which name is Average pooled, means $\bar{M}$. When we use average pooling, we use $\left\lfloor \frac{H}{n}\right\rfloor$ for kernel size and stride and not padding. Then The size of $\bar{M}$ is n×n. We compute the location with the largest and smallest values in $\bar{M}$ using the following equation:

(4) $$\begin{array}{c} {\bar{i}}_{m},{\bar{j}}_{m}=\underset{i,j}{\mathrm{argmax}}{\bar{M}}_{ij},\\ {\bar{i}}_{l},{\bar{j}}_{l}=\underset{i,j}{\mathrm{argmin}}{\bar{M}}_{ij}. \end{array}$$

To convert the coordinates to the original coordinate in an image, we compute $i_{m}={\bar{i}}_{m}\times \frac{W}{n},j_{m}={\bar{j}}_{m}\times \frac{H}{n}$, $i_{l}={\bar{i}}_{l}\times \frac{W}{n},j_{l}={\bar{j}}_{l}\times \frac{H}{n}$. We uniformly and randomly select a square patch $P_{m}$ that includes the area $R_{m}$ from $\left(i_{m},\;j_{m}\right)$ to $\left(i_{m}+\frac{W}{n},\;j_{m}+\frac{H}{n}\right)$. In other words, $P_{m}$ represents the wider area than $R_{m}$, where $R_{m}\subset P_{m}$. We use a parameter *r* to determine the patch size of o×o using o=⌊W×r⌋.

**Figure 3:**
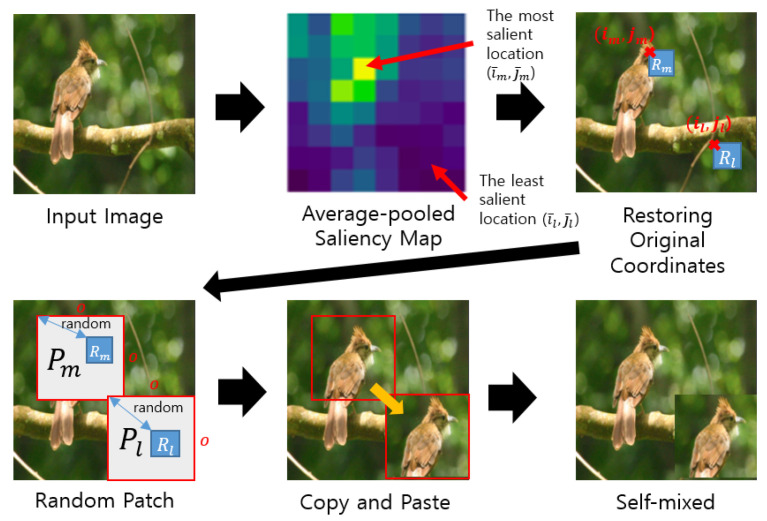
Procedure of SalfMix. The saliency map calculated by Equation (2), next, average-pooled image is created through an average pooling process. Then, the most and least salient regions are detected in the average-pooled image. Patches $P_{m}$ and $P_{l}$ are randomly chosen, and the patches must include the detected regions $R_{m}$ and $R_{l}$. Finally, the pixel values in $P_{m}$ are copied to the region of $P_{l}$ in the same image.

$P_{l}$ is selected via the same way, except for using $i_{l}$ and $j_{l}$ instead of $i_{m}$ and $j_{m}$. The region $R_{l}$ is defined from $\left(i_{l},\;j_{l}\right)$ to $\left(i_{l}+\frac{W}{n},\;j_{l}+\frac{H}{n}\right)$. Finally, we replace patch $P_{l}$ with patch $P_{m}$. The procedure of SalfMix is demonstrated in Figure 3. Algorithm 1 shows the final algorithm of SalfMix. The *f* is the model to be trained, and it is used to calculated the saliency map through Equation (2) of line 4 of Algorithm 1. We obtain the self-mixed image $\hat{x}$ using this algorithm.
**Algorithm 1** SalfMix1:**Input:** f,x,y,n,r  // *training image, label, parameters*2:**Output:** $\hat{x}$  // *self-mixed image*3: 4:M← Compute saliency map using *x* and *y* in Equations (2) and (3).5:$\bar{M}\leftarrow$ Perform an average pooling to produce n×n image.6:$P_{m},\;P_{l}\leftarrow$ Obtain the most and least salient patches with *r*.7:$\hat{x}\leftarrow$ Replace $P_{l}$ with $P_{m}$ in input image *x*.8:**return** $\hat{x}$

### 4.4. HybridMix

A self-mixed image $\hat{x}$ was generated through the SalfMix process in Section 4.3. The SalfMix can be integrated easily with two images-based data augmentation approaches, such as Mixup, SaliencyMix, and CutMix, as shown in Equation (1). Consequently, we present three types of HybridMix as shown in Table 2.

Finally, A training algorithm with Hybridmix is shown in Algorithm 2. In this algorithm, $X=\{X_{1},\ldots ,X_{N_{b}}\}$ and $Y=\{Y_{1},\ldots ,Y_{N_{b}}\}$ denote the total training set where $X_{1},\ldots ,X_{N_{b}}$ and $Y_{1},\ldots ,Y_{N_{b}}$ represent each mini-batch of training data and labels, respectively. The number of mini-batches is $N_{b}$. Each mini-batch $X_{i}=\left\{X_{i1},\ldots ,X_{iN_{d}}\right\}$ and $Y_{i}=\left\{Y_{i1},\ldots ,Y_{iN_{d}}\right\}$ for arbitrary *i* where $N_{d}$ is the number of examples in each mini-batch. $N_{e}$ is the total number of epochs. In line 8 of the Algorithm 2, *z* is an integer value to represent the index of another image to be mixed.In line 9 of the Algorithm 2, *i* is each index in mini-batch. id means the *d*-th data of the *i*-th mini-batch, and iz means the *z*-th data of the *i*-th mini-batch, where *z* is randomly sampled index.
**Algorithm 2** Training with HybridMix1:**Input:** X, Y, *f*, *n*, *r* // *training set, CNN, parameters*2: 3:**for** $e=1,\ldots ,N_{e}$ **do** // *epoch*4:    **for** $i=1,\ldots ,N_{b}$ **do** // *mini-batch*5:        ${\tilde{X}}_{i},{\tilde{Y}}_{i}\leftarrow X_{i},Y_{i}$6:        **if** e>1 **then**7:           **for** $d=1,\ldots ,N_{d}$ **do** // *data in a mini-batch*8:               z← Randomly sampled within [$1,N_{d}$]9:               $x_{1},y_{1},x_{2},y_{2}\leftarrow X_{id},Y_{id},X_{iz},Y_{iz}$10:               ${\hat{x}}_{1}=\mathrm{SalfMix}\left(f,x_{1},y_{1},n,r\right)$11:               ${\hat{x}}_{2}=\mathrm{SalfMix}\left(f,x_{2},y_{2},n,r\right)$12:               $\tilde{x},\tilde{y}=T_{t}\left({\hat{x}}_{1},y_{1},{\hat{x}}_{2},y_{2}\right)$13:               ${\tilde{X}}_{id},{\tilde{Y}}_{id}\leftarrow \tilde{x},\tilde{y}$14:           **end for**15:        **end if**16:        Optimizing *f* using ${\tilde{X}}_{i}$ and ${\tilde{Y}}_{i}$ via backpropagation.17:    **end for**18:**end for**

## 5. Experiments and Results

### 5.1. Experimental Settings

**Dataset.** To demonstrate the effectiveness of our proposed method, we evaluated the proposed method on multiple datasets such as CIFAR-10, CIFAR-100, TinyImageNet-200, and VOC 2007/2012 datasets [14,29,30]. The CIFAR-10, CIFAR-100, and TinyImageNet-200 dataset are used for evaluating classification performance, and the VOC 2007/2012 datasets are used for evaluating object detection performance. The CIFAR-10 and CIFAR-100 each comprise 50 K training images and 10 K test images, and each image has the same size as 32×32. The numbers of classes in CIFAR-10 and CIFAR-100 are 10 and 100, respectively. TinyImageNet-200, which is a smaller dataset than the original ImageNet dataset [14] with fewer image classes (e.g., 200), comprises 100 K training images and 10 K test images, and each image has the same size as 64×64. VOC 2007/2012 dataset contains 20 object categories, and we used 16 K and 1.2 K images for train and test, respectively.The summary of dataset is shown in Table 3 below. 

**Implementation.** Our code was implemented using PyTorch [31] for the classification task and Detectron2 [32] for the detection. The experiments were conducted on a Quadro RTX 8000 GPUs for classification tasks and four A100 GPUs for object detection tasks. 

**Parameter Settings for Classification Task.** We used PreActResNet-18, PreActResNet-50, and PreActResNet-101 as baseline architectures for classification experiments [33]. The number means the number of layers of the architecture. (e.g., PreActResNet-50 means the architecture has 50 layers.) The number of parameters for each model are 11 M, 24 M, and 43 M, respectively. We trained the models for 300 epochs.

On the CIFAR [29] datasets, the initial learning rate was 0.05, and the learning rate was multiplied by 0.1 at 150 and 225 epochs. On the TinyImageNet-200 [34], the initial learning rate was the same as for CIFAR, but it was multiplied by 0.1 at 75, 150, and 225 epochs. The mini-batch size was set to 64 and 128 for CIFAR and TinyImageNet-200, respectively. Stochastic gradient descent was used with a momentum of 0.9 and weight decay of $1\times 10^{-4}$. The size of the saliency map that passed average pooling was 4×4 and 8×8 for CIFAR and TinyImageNet-200, respectively. The patch size of $P_{m}$ and $P_{l}$ were set according to r=0.3.

The parameter values for each data augmentation method were set as the default values used in their corresponding studies [16,18,19,22]. Cutout uses regional dropout, and its mask size is set to 0.5 ratio of the input image size. Mixup, CutMix, and SaliencyMix use the same beta distribution as a ratio to mix two images. In ResizeMix, α and β to determine the image patch size were set to 0.1 and 0.8, respectively. For geometric data augmentation, random resized crop, random horizontal flip and normalization were used for the CIFAR dataset, and additional color jittering and brightness were used for TinyImageNet-200. 

**Parameter Settings for Detection Task.** For the object detection task, ResNet architecture with 50 layers was used. We trained the ResNet on the TinyImageNet-200 dataset, and the experimental setting is the same as the TinyImageNet-200 experiment for classification tasks. We transferred it to the detection task of VOC 2007/2012 datasets. Parameters for batch size, learning rate, and training iterations were set to 8, 0.02 and 24 k, respectively. The learning rate was multiplied by 0.1 at 18 k and 22 k iterations.

### 5.2. CIFAR-10/100 Classification

The experimental results are shown in Table 4 and Table 5. We measured the Top-1 error rates to compare with the competing methods. HybridMix v1, HybridMix v2, and HybridMix v3 outperform state-of-the-art data augmentation approaches, such as Mixup, SaliencyMix, and CutMix. Particularly, HybridMix v3 achieved 3.38%, 2.89% and 2.75% error rates, respectively, when using PreActResNet-18, 50, and 101 models on the CIFAR-10 dataset. It reduced 1.79%, 1.71%, and 1.74% error rates, respectively, compared to the baseline and showed 0.58%, 0.18%, and 0.2% performance improvements compared to CutMix. It also showed better performance than most recently proposed techniques such as SaliencyMix and ResizeMix.

On the CIFAR-100 dataset, our method also achieves performance improvements at all depths of the model, as shown in Table 5. The state-of-the-art on PreActResNet-18 was an error rate of 20.02% achieved by SaliencyMix. HybridMix v2 outperformed with an error rate of 19.88%. Additionally, we conducted additional experiments using the same parameter settings in the PuzzleMix paper [21]. HybridMix v1, v2, and v3 achieved 19.37%, 19.09%, and 18.96% error rates, respectively, leading to 0.66% performance improvements using HybridMix v3 compared to PuzzleMix.

We also conduct additional experiments using other architectures. PyramidNet-110 [35] and RegNet-200M [36] were used, and the number of parameters are 1.7 and 2.3 million, respectively. The experimental settings for PyramidNet-110 are the same as the CutMix paper [19]. Furthermore, the settings for RegNet-200M were adopted from the source code (https://github.com/yhhhli/RegNet-Pytorch, accessed on 1 August 2021).

As a results, HybridMix v3 achieved 17.65% and 24.99% using PyramidNet-110 and RegNet-200M, respectively. Especially, HybridMix v3 significantly reduces the error rates compared to the RegNet-200M baseline model (29.44%, Baseline VS 24.99%, HybridMix v3), as shown in Table 6.

### 5.3. TinyImageNet-200 Classification

Table 7 depicts the experimental results on the TinyImageNet-200 dataset. The largest performance improvement was achieved with PreActResNet-101, which is the deepest architecture. This is an improvement of 7.33% from the baseline and 1.58% from CutMix using HybridMix v3. Similarly, HybridMix v1 and HybridMix v2 showed 2.29% and 0.58% performance improvements over Mixup and SaliencyMix, respectively.

Similar to the experiment on CIFAR-100 dataset, we also compared to PuzzleMix using the same settings in the PuzzleMix paper. HybridMix v1, v2, and v3 recorded 35.52%, 34.25%, and 33.75%, respectively. These are 1%, 2.27%, and 2.77% performance improvements over Puzzle Mix.

### 5.4. Transferring to Object Detection Task

We used the ResNet50 model trained with HybridMix v1, v2, and v3 as the backbone of Faster R-CNN [37]. Data augmentation techniques were used only to train the backbone, ResNet50. ResNet50 models are pre-trained on the TinyImageNet-200 dataset and used the pre-trained models as backbone networks for Faster R-CNN. The Faster R-CNN was fine-tuned on VOC 2007/2012 training data. To confirm the effect of HybridMix on object detection, ResNet models were independently trained using other two images-based augmentation approaches, such as Mixup, SaliencyMix, CutMix, and ResizeMix [18,19,20,22]. We used mean average precision (mAP) as the evaluation metric. ${\mathrm{mAP}}_{50}$ and ${\mathrm{mAP}}_{75}$ mean mAP were calculated at IoU = 0.5 and IoU = 0.75, respectively.

Table 8 shows the object detection experimental results on VOC 2007/2012 dataset. HybridMix-trained backbone network showed 1.47%, 3.65%, and 4.52% performance improvements in terms of ${\mathrm{mAP}}_{50}$, compared to the baseline, respectively. Each version of HybridMix also showed performance improvements over other two images-based data augmentations. HybridMix v3 which is a combination of CutMix and SalfMix led to 1.34% performance improvement over CutMix, and it has the best performance in terms of ${\mathrm{mAP}}_{50}$. Compared to the Cutout-trained model, the SalfMix-trained model showed a 0.89% performance improvement in terms of ${\mathrm{mAP}}_{50}$. This means that copying and pasting in a single image is effective for improving the object detection accuracies.

## 6. Ablation Study

### 6.1. Visualization of Training Graph

**Figure 4:**
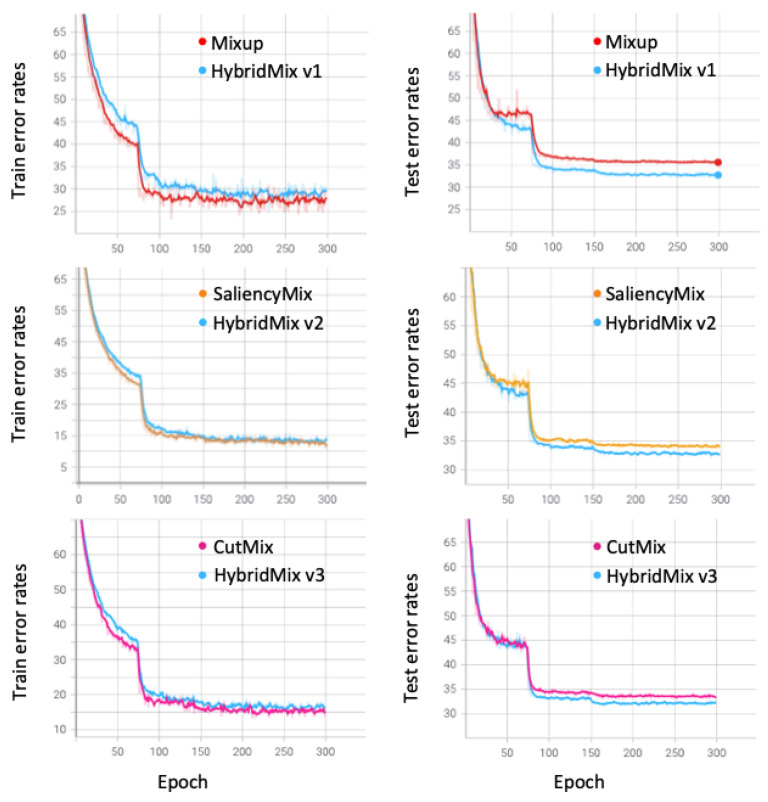
Top-1 classification error rates for two images-based data augmentation (Mixup [18], SaliencyMix [20], and CutMix [19]) and HybridMix v1, v2, and v3 on TinyImageNet-200 with PreActResNet-50.

We visualize training graphs on TinyImageNet-200 experiments performed in Section 5.3, as shown in Figure 4. The graphs were based on the PreActResNet-50 model, and we compared HybridMix v1, v2, and v3 with two images-based data augmentation approaches such as Mixup, SaliencyMix and CutMix, respectively. Interestingly, error rates of HybridMix for training data are higher than the error rates of Mixup and CutMix, but the HybridMix v1 and v3 have achieved low test error rates. The high training error rates were caused by adding the SalfMix process, and it provides better generalization capability.

### 6.2. Effectiveness of the Saliency Map

Cutout [16], which is a representative single image-based data augmentation approach, performs regional dropout. When Cutout drops a region of an image, the algorithm does not consider its saliency. We compared Cutout with and without the saliency map to show the effectiveness of the saliency map. We obtained a saliency map from an image and dropped out the least salient region $P_{l}$. Table 9 shows the experimental results using PreActResNet-101. Interestingly, the saliency map is also effective in improving Cutout. In other words, Cutout showed better performance with the saliency map than without the saliency map. It achieves 0.11%, 0.59% and 0.31% performance improvements on the CIFAR-10, CIFAR-100, and TinyImageNet-200 datasets, respectively. Similarly, we experimented with the effectiveness of the saliency map in SalfMix. Original SalfMix replaces the $P_{m}$ patch with the $P_{l}$ patch using the saliency map. For SalfMix without the saliency map, $P_{m}$ and $P_{l}$ are randomly selected. SalfMix achieved 0.07%, 0.81%, and 1.86% performance improvements on the CIFAR-10, CIFAR-100 and TinyImageNet-200 datasets, respectively.

### 6.3. Effectiveness of SalfMix

We demonstrated better performance by dropping the least salient region than by randomly dropping a region in Cutout. This means that salient regions are important for training and it is better to remove the least salient regions during data augmentation. The drawback of Cutout is that some features are filled with zeros or random values, whereas SalfMix copies and fills this dropped region with the most salient region. We compared the Top-1 classification error rates when using Cutout with a saliency map and SalfMix on the three datasets, and the results are listed in Table 9. SalfMix achieved performance improvements of 0.05%, 0.98%, and 2.16% compared to Cutout with a saliency map, on CIFAR-10, 100, and TinyImageNet-200, respectively.

### 6.4. Hyperparameter *r*

**Figure 5:**
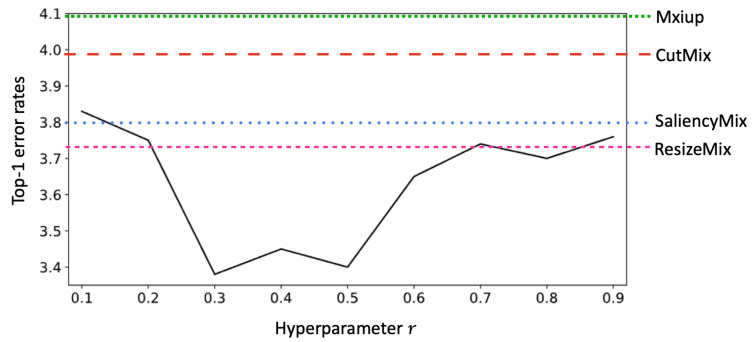
Top-1 classification error rates for HybridMix v3 with varying *r* on CIFAR-10 with PreActResNet-18.

Hyperparameter *r* is important for determining the performance of the proposed method. We varied the hyperparameter *r*, which determines the patch size, on CIFAR-10. Varying *r* in the interval [0.1, 0.9] with increments of 0.1, the best performance was achieved when *r* is within 0.3 and 0.5 intervals, as shown in Figure 5. The experiment has shown that our method can achieve better performance than CutMix, Mixup, SaliencyMix, and ResizeMix, which are state-of-the-art data augmentation techniques, without carefully choosing *r*.

### 6.5. Effectiveness of Average Pooling

When SalfMix is applied to the input image during training, a saliency map is obtained. Then, we apply average pooling to a certain size (e.g., 4×4 or 8×8 ), as shown in Figure 2. When we find ($i_{m}$, $j_{m}$) and ($i_{l}$, $j_{l}$) in the SalfMix process, we compare and analyze the proposed method with the saliency map using and not using average pooling.

The experimental setup is the same as in Section 5.1, and the datasets used in the experiments are CIFAR-10, CIFAR-100, and TinyImageNet-200. We used HybridMix v3 and PreActResNet-101 for experiments. The size of saliency map without average pooling is 32×32 for CIFAR datasets, 64×64 for TinyImageNet-200, and the SalfMix process is the same afterward. Experimental results showed that the method using average pooling performed better on the CIFAR-10, CIFAR-100, and TinyImageNet-200 with a difference of 0.05%, 0.11% and, 0.64%, respectively, as shown in Table 10.

The regions of the highest score and lowest score are found in pixels because the saliency map withoutaverage pooling is the same size as the image. However, using the saliency map with average pooling, we can find the regions of the highest score and lowest score. This is considered to be a better performance because it smoothens the saliency map.

### 6.6. Visualizing Augmented Data

**Figure 6:**
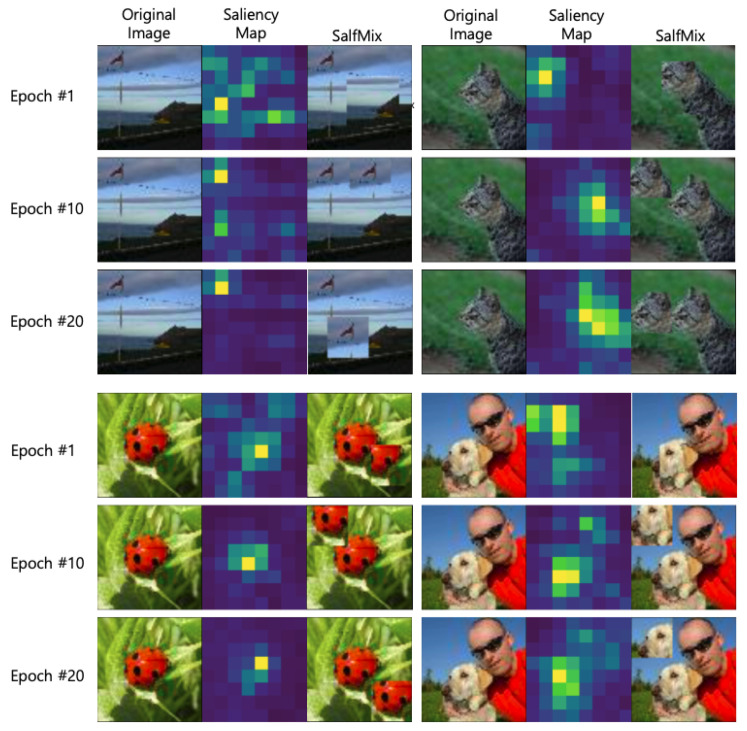
Visualization of augmented data on TinyImageNet-200. The original image size is 64×64. The saliency map becomes more accurate according to the epoch.

In the case of SalfMix, images are randomly mixed at the beginning of training because the saliency map is not accurate. Based on the visualization of augmented data, we can find a relatively accurate saliency map after a small number of epochs (e.g., e<10), as shown in Figure 6. For the flagpole image in the first, second, and third columns, SalfMix randomly selected the patches at epoch #1. After 10 epochs, the part containing a flagpole was correctly detected as the most salient region. Similarly, for the second image shown in the fourth, fifth, and sixth columns, the extracted region was not good at epoch #1, but after a few epochs, SalfMix could estimate the saliency map accurately. For the two labels lady bug and labrador retriever, the tendency was similar.

## 7. Conclusions and Future Works

In this paper, we proposed a novel single image-based data augmentation technique using a saliency map. The proposed technique replaces the least salient region with the most salient region in a single image during a training process. Through several ablation studies, we have proved the effectiveness of the saliency map and SalfMix. Finally, we showed that SalfMix outperforms Cutout, which is one of state-of-the-art single image-based data augmentation techniques, on the CIFAR-10, CIFAR-100, TinyImageNet-200, and VOC 2007/2012 datasets. Furthermore, the HybridMix technique, which combines SalfMix with two images-based data augmentation strategies, achieved performance improvements on three datasets, such as CIFAR-10, CIFAR-100, and TinyImageNet-200. Particularly, HybridMix v3 using PreActResNet-101 showed state-of-the-art performance on the CIFAR-10, CIFAR-100, and TinyImageNet-200 datasets, respectively. Additionally, the HybridMix v3-trained model led to a performance improvement by 1.34% in terms of ${\mathrm{mAP}}_{50}$ over CutMix on the VOC 2007/2012 dataset. Our future work will be considering to improve the training speed and also experiment on segmentation tasks.

## Figures and Tables

**Table 1:** Key differences among state-of-the-art data augmentation techniques.

Approach	Mixed?	Single-Image?	Saliency?
Cutout [16]	✘	✔	✘
Mixup [18]	✔	✘	✘
CutMix [19]	✔	✘	✘
SaliencyMix [20]	✔	✘	✔
SnapMix [23]	✔	✘	✔
ResizeMix [22]	✔	✘	✘
PuzzleMix [21]	✔	✘	✔
Self-Augmentation [24]	✔	✔	✘
**SalfMix**	✔	✔	✔
**HybridMix**	✔	Both	✔

**Table 2:** Three types of HybridMix.

Version	Two-Image-Based Mix Function $T_{t}$
HybridMix v1	Mixup ($x_{1}$, $y_{1}$, $x_{2}$, $y_{2}$)
HybridMix v2	SaliencyMix ($x_{1}$, $y_{1}$, $x_{2}$, $y_{2}$)
HybridMix v3	CutMix ($x_{1}$, $y_{1}$, $x_{2}$, $y_{2}$)

**Table 3:** Summary of Dataset for classification and object detection task. Num of Images means the sum of the number of training images and test images.

Dataset	Resolution	Num of Images	Num of Classes
CIFAR-10	32 × 32	60 K	10
CIFAR-100	32 × 32	60 K	100
TinyImageNet-200	64 × 64	110 K	200
VOC 2007/2012	-	17.2 K	20

**Table 4:** Comparison of Top-1 classification error rates (%) for Baseline, Cutout, SalfMix, Mixup, SaliencyMix, CutMix, ResizeMix, and HybridMix on CIFAR-10.

	PreActResNet-18	PreActResNet-50	PreActResNet-101
Baseline	5.17 ± 0.27	4.6 ± 0.2	4.49 ± 0.18
+Cutout [16]	4.3 ± 0.09	3.77 ± 0.08	3.54 ± 0.11
+SalfMix	**4.14** ± **0.25**	**3.61** ± **0.09**	**3.38** ± **0.11**
+Mixup [18]	4.1 ± 0.39	3.56 ± 0.04	3.54 ± 0.08
+SaliencyMix [20]	3.8 ± 0.1	2.98 ± 0.1	2.82 ± 0.08
+CutMix [19]	3.96 ± 0.21	3.07 ± 0.09	2.95 ± 0.06
+ResizeMix [22]	3.74 ± 0.2	3.09 ± 0.11	2.85 ± 0.09
+HybridMix v1	3.85 ± 0.13	3.22 ± 0.04	3.04 ± 0.14
+HybridMix v2	3.74 ± 0.05	2.94 ± 0.09	2.78 ± 0.04
+HybridMix v3	**3.38** ± **0.07**	**2.89** ± **0.11**	**2.75** ± **0.07**

**Table 5:** Comparison of Top-1 classification error rates (%) for Baseline, Cutout, SalfMix, Mixup, SaliencyMix, CutMix, ResizeMix, PuzzleMix, and HybridMix on CIFAR-100. (1200) means the number of epochs.

	PreActResNet-18	PreActResNet-50	PreActResNet-101
Baseline	24.22 ± 0.22	22.02 ± 0.18	21.81 ± 0.24
+Cutout [16]	23.72 ± 0.27	21.64 ± 0.43	21.46 ± 0.25
+SalfMix	**22.64** ± **0.13**	**20.48** ± **0.17**	**19.89** ± **0.13**
+Mixup [18]	21.78 ± 0.4	18.91 ± 0.26	18.82 ± 0.37
+SaliencyMix [20]	20.02 ± 0.13	17.5 ± 0.16	17.33 ± 0.09
+CutMix [19]	20.51 ± 0.17	17.72 ± 0.17	17.61 ± 0.25
+ResizeMix [22]	20.96 ± 0.11	17.56 ± 0.09	17.36 ± 0.19
+HybridMix v1	21.42 ± 0.17	18.27 ± 0.12	17.45 ± 0.12
+HybridMix v2	19.88 ± 0.27	17.38 ± 0.27	**17.22** ± **0.21**
+HybridMix v3	**19.84** ± **0.09**	**17.3** ± **0.25**	17.25 ± 0.23
+PuzzleMix (1200) [21]	19.62	-	-
+HybridMix v1 (1200)	19.37 ± 0.18	-	-
+HybridMix v2 (1200)	19.09 ± 0.13	-	-
+HybridMix v3 (1200)	**18.96** ± **0.13**	-	-

**Table 6:** Impact of HybridMix v3 using other architectures on CIFAR-100. Err. means error rates.

Model	# Params	Top-1 Err. (%)	Top-5 Err. (%)
PyramidNet-110 ($\tilde{\alpha }=64$) [35]	1.7 M	19.85	4.66
PyramidNet-110 + CutMix [19]	1.7 M	17.97	3.83
PyramidNet-110 + HybridMix v3	1.7 M	**17.65**	**3.71**
RegNet-200M [36]	2.3 M	29.44	9.7
RegNet-200M + CutMix [19]	2.3 M	26.04	8.07
RegNet-200M + HybridMix v3	2.3 M	**24.99**	**7.58**

**Table 7:** Comparison of Top-1 classification error rates (%) for Baseline, Cutout, SalfMix, Mixup, SaliencyMix, CutMix, ResizeMix, PuzzleMix, and HybridMix on TinyImageNet-200. (1200) means the number of epochs.

	PreActResNet-18	PreActResNet-50	PreActResNet-101
Baseline	42.33 ± 0.21	38.58 ± 0.24	38.04 ± 0.1
+Cutout [16]	42.04 ± 0.31	38.36 ± 0.21	37.96 ± 0.36
+SalfMix	**40.28** ± **0.28**	**35.92** ± **0.07**	**35.49** ± **0.08**
+Mixup [18]	40.22 ± 0.2	35.51 ± 0.15	35.05 ± 0.47
+SaliencyMix [20]	37.76 ± 0.05	32.83 ± 0.47	31.82 ± 0.15
+CutMix [19]	38.11 ± 0.32	33.54 ± 0.19	32.29 ± 0.28
+ResizeMix [22]	38.47 ±0.25	33.25 ± 0.12	32.16 ± 0.17
+HybridMix v1	38.46 ± 0.14	33.52 ± 0.11	32.76 ± 0.3
+HybridMix v2	37.52 ± 0.26	32.19 ± 0.21	31.24 ± 0.12
+HybridMix v3	**36.86 ± 0.17**	**31.77± 0.06**	**30.71 ± 0.12**
+PuzzleMix (1200) [21]	36.52	-	-
+HybridMix v1 (1200)	35.52 ± 0.21	-	-
+HybridMix v2 (1200)	34.25 ± 0.13	-	-
+HybridMix v3 (1200)	**33.75** ± **0.25**	-	-

**Table 8:** Comparison of transfer learning performance to object detection task using various data augmentation strategies, such as Cutout, SalfMix, Mixup, SaliencyMix, CutMix, ResizeMix, and HybridMix.

Backbone Network	Faster-RCNN		
	mAP (%)	${\mathrm{mAP}}_{50}$ (%)	${\mathrm{mAP}}_{75}$ (%)
ResNet50 (Baseline)	37.04	65.86	36.69
Cutout-trained [16]	36.9	65.4	36.74
SalfMix-trained	**37.23**	**66.29**	**36.85**
Mixup-trained [18]	37.21	65.63	36.23
SaliencyMix-trained [20]	40.31	69.46	40.71
CutMix-trained [19]	39.72	69.04	39.9
ResizeMix-trained [22]	39.2	68.05	39.23
HybridMix v1-trained	38.66	67.33	38.21
HybridMix v2-trained	40.45	69.51	**40.87**
HybridMix v3-trained	**40.78**	**70.38**	40.64

**Table 9:** Comparison of Top-1 classification error rates (%) for Cutout and SalfMix with and without the saliency map when using PreActResNet-101. Sal. means saliency map.

Dataset	Cutout	Cutout w/Sal.	SalfMix w/o Sal. (The Method Is Similar to the Method Shown in the Paper [24])	SalfMix
CIFAR-10	3.54	3.43	3.45	**3.38**
CIFAR-100	21.46	20.87	20.7	**19.89**
TinyImageNet-200	37.96	37.65	37.35	**35.49**

**Table 10:** Comparison of Top-1 classification error rates (%) for HybridMix v3 without average pooling and with average pooling using PreActResNet-101.

Dataset	w/o Average Pooling	w/Average Pooling
CIFAR-10	2.8	**2.75**
CIFAR-100	17.36	**17.25**
TinyImageNet-200	31.35	**30.71**

## Data Availability

Not applicable.
